# Distinct airway epithelial immune responses after infection with SARS-CoV-2 compared to H1N1

**DOI:** 10.1038/s41385-022-00545-4

**Published:** 2022-07-15

**Authors:** Helen Stölting, Laury Baillon, Rebecca Frise, Katie Bonner, Richard J. Hewitt, Philip L. Molyneaux, Mindy L. Gore, Breathing Together Consortium, Steve Turner, Adnan Custovic, Peter Ghazal, Jonathan Grigg, Mindy Gore, Raquel Granell, Benjamin Marsland, Ultan F. Power, Graham Roberts, Sejal Saglani, Jürgen Schwarze, Michael Shields, Andrew Bush, Wendy S. Barclay, Sejal Saglani, Clare M. Lloyd

**Affiliations:** 1 National Heart and Lung Institute, Imperial College London, London, UK; 2 Department of Infectious Disease, Imperial College London, London, UK; 3 Chelsea and Westminster Hospital Foundation Trust, London, UK; 4 Royal Brompton and Harefield Hospitals, Guy’s and St Thomas’ NHS Foundation Trust, London, UK; 5 Child Health, University of Aberdeen, Aberdeen, AB25 2ZG UK; 6 Department of Paediatrics, Imperial College and Royal Brompton Hospital, London, SW3 6NP UK; 7 Division of Infection and Pathway Medicine, Deanery of Biomedical Sciences, University of Edinburgh Medical School, Edinburgh, EH16 4TJ UK; 8 Centre for Child Health, Blizard Institute, Queen Mary University of London, London, E1 2AT UK; 9 MRC Integrative Epidemiology Unit, Population Health Sciences, Bristol Medical School, University of Bristol, Bristol, BS8 2BN UK; 10 Department of Immunology and Pathology, Monash University, Melbourne, VIC 3004 Australia; 11 Wellcome-Wolfson Institute for Experimental Medicine, School of Medicine, Dentistry and Biomedical Sciences, Queen’s University Belfast, Belfast, BT9 7BL UK; 12 Clinical and Experimental Sciences and Human Development and Health, Faculty of Medicine, University of Southampton, Southampton, SO17 1BJ UK; 13 NIHR Southampton Respiratory Biomedical Research Unit, University Hospital Southampton NHS Foundation Trust, Southampton, SO16 6YD UK; 14 The David Hide Asthma and Allergy Research Centre, St Mary’s Hospital, Newport, Isle of Wight, Trust, PO30 5TG UK; 15 Child Life and Health, Centre for Inflammation Research, Queen’s Medical Research Institute, University of Edinburgh, Edinburgh, EH16 4TJ UK

## Abstract

Children are less likely than adults to suffer severe symptoms when infected with severe acute respiratory syndrome coronavirus 2 (SARS-CoV-2), while influenza A H1N1 severity is comparable across ages except for the very young or elderly. Airway epithelial cells play a vital role in the early defence against viruses via their barrier and immune functions. We investigated viral replication and immune responses in SARS-CoV-2-infected bronchial epithelial cells from healthy paediatric (*n* = 6; 2.5–5.6 years old) and adult (*n* = 4; 47–63 years old) subjects and compared cellular responses following infection with SARS-CoV-2 or Influenza A H1N1. While infection with either virus triggered robust transcriptional interferon responses, including induction of type I (*IFNB1*) and type III (*IFNL1*) interferons, markedly lower levels of interferons and inflammatory proteins (IL-6, IL-8) were released following SARS-CoV-2 compared to H1N1 infection. Only H1N1 infection caused disruption of the epithelial layer. Interestingly, H1N1 infection resulted in sustained upregulation of SARS-CoV-2 entry factors *FURIN* and *NRP1*. We did not find any differences in the epithelial response to SARS-CoV-2 infection between paediatric and adult cells. Overall, SARS-CoV-2 had diminished potential to replicate, affect morphology and evoke immune responses in bronchial epithelial cells compared to H1N1.

## Introduction

Severe acute respiratory syndrome coronavirus 2 (SARS-CoV-2) is the causative agent of coronavirus disease 19 (COVID-19), a global pandemic that originated in Wuhan, China, at the end of 2019 and has resulted in over 6 million deaths to date (according to WHO, as of March 2022). While H1N1pdm09, the influenza A virus responsible for the 2009 pandemic, can cause severe disease in both children and adults^1^, detrimental outcomes of COVID-19 are more prominent in adults, with roughly 20% having developed severe or critical disease during the first wave of COVID-19^2,3^. In contrast, SARS-CoV-2-infected children typically remain asymptomatic or experience mild to moderate symptoms, and severe disease or fatality are rare^4–6^. Some studies propose that, in addition to decreased severity, susceptibility to infection is also reduced in children compared to adults^7–9^, but the potential mechanisms underlying these discrepancies remain poorly understood. Entry of SARS-CoV-2 into host cells is dependent on angiotensin converting enzyme 2 (ACE2), which acts as a receptor for SARS-CoV-2 Spike protein, and the protease transmembrane protease serine 2 (TMPRSS2)^10^. In addition, other host factors, such as cathepsin L, furin proteases and neuropilins, have been ascribed important roles in the pre-activation and entry of SARS-CoV-2^11–15^. Decreased expression of SARS-CoV-2 entry-related genes in children compared to adults has been described^16,17^, offering a potential explanation for differing susceptibility to infection, but reports are conflicting and differences in the expression of these host factors between children and adults have not been confirmed in other studies^18–21^.

The airway epithelium acts as an important first line of defence against respiratory viruses, not only by providing a physical barrier between the external environment and the internal milieu, but also via the release of antiviral and pro-inflammatory mediators^22^. Airway epithelial cells express a range of pattern recognition receptors (PRRs), such as the endosomal Toll-like receptors (TLRs) TLR3, TLR7, TLR8 and TLR9 and the cytoplasmic receptors retinoic acid-inducible gene I (RIG-I) and melanoma differentiation-associated gene 5 (MDA5), which allow them to sense viruses and initiate the antiviral interferon response. Upon viral recognition, airway epithelial cells constitute an early source of type I and III interferons (IFNs), which act to rapidly establish an antiviral state via the induction of hundreds of interferon-stimulated genes (ISGs). In particular, type I IFNs are crucial in the successful defence against respiratory viruses such as influenza virus^23^, and mounting evidence suggests that the early type I IFN response is also central to the response to SARS-CoV-2^24–26^.

In addition to their role in initiating antiviral responses, both nasal and bronchial epithelial cells are also considered primary portals of entry for SARS-CoV-2^27^, not least due to their expression of both ACE2 and TMPRSS2^28^. Air–liquid interface (ALI) cultures of primary airway epithelial cells are an ideal model for the study of viral infections, as they form a polarised, highly differentiated mucociliary layer representative of the in vivo airway wall^29^ and retain donor disease characteristics such as age or disease status^30–32^. Accordingly, ALI cultures have been widely used to investigate responses to SARS-CoV-2 infection^15,33–36^. However, a detailed characterisation of SARS-CoV-2 infection in paediatric airway epithelial cells compared to adult cells is lacking. While some groups have also used infection with H1N1 as a comparator for SARS-CoV-2^36–38^, an approach that has revealed differences in the interferon induction between the two viruses, these studies have been restricted to analysis of gene expression and early time points. We hypothesised that the disparate severity of COVID-19 in children and adults could be explained by intrinsic differences in airway epithelial cells from these groups and that SARS-CoV-2 and H1N1 would evoke distinct responses in these cells We infected ALI cultures of primary bronchial epithelial cells from healthy children (*n* = 6) and adults (*n* = 4) with an early-lineage clinical isolate of SARS-CoV-2 (England/IC19/2020) or the pandemic strain of H1N1 (A/England/195/2009) to directly compare susceptibility to infection and epithelial antiviral immune responses according to age and virus. We monitored productive infection using plaque assays and immunofluorescence staining for SARS-CoV-2 nucleocapsid protein up until 9 days post-infection (dpi). Interferon and inflammatory responses were assessed at mRNA and protein level, and we measured gene expression levels of SARS-CoV-2 entry-related factors and receptors involved in SARS-CoV-2 recognition both at baseline and in response to viral infection.

## Results

### Bronchial epithelial cells from paediatric and adult donors displayed comparable cellular composition and SARS-CoV-2 entry factor expression

We aimed to study whether differences in COVID-19 susceptibility and severity between children and adults might be explained by intrinsically disparate epithelial cell responses to infection. To this end, we collected bronchial epithelial cells from six healthy children (median age 4.6 years) and four healthy adults (median age 52.5 years; Table 1). There were no differences between nasal and bronchial epithelial cells from paediatric donors with respect to cell morphology, replication of SARS-CoV-2 or interferon response to infection (Supplementary Fig. 1A–C), and therefore bronchial epithelial cells were used as a model of the airway epithelium throughout this study.

**Table 1:** Donor demographics.

	Paediatric (*n* = 6)	Adult (*n* = 4)
Median age (range)	4.6 (2.5–5.6)	52.5 (47–63)
Male/female	3/3	2/2
Ethnicity (%)		
Asian	1 (17%)	2 (50%)
White Caucasian	4 (67%)	2 (50%)
Mixed Black/White Caucasian	1 (17%)	–
Atopy status (%)		
Atopic	3 (50%)	–
Non-Atopic	3 (50%)	–
Unknown	–	4 (100%)
Smoking status (%)		
Current smoker		0 (−)
Ex-smoker	n/a	2 (50%)
Never smoker		2 (50%)

**Fig. 1:**
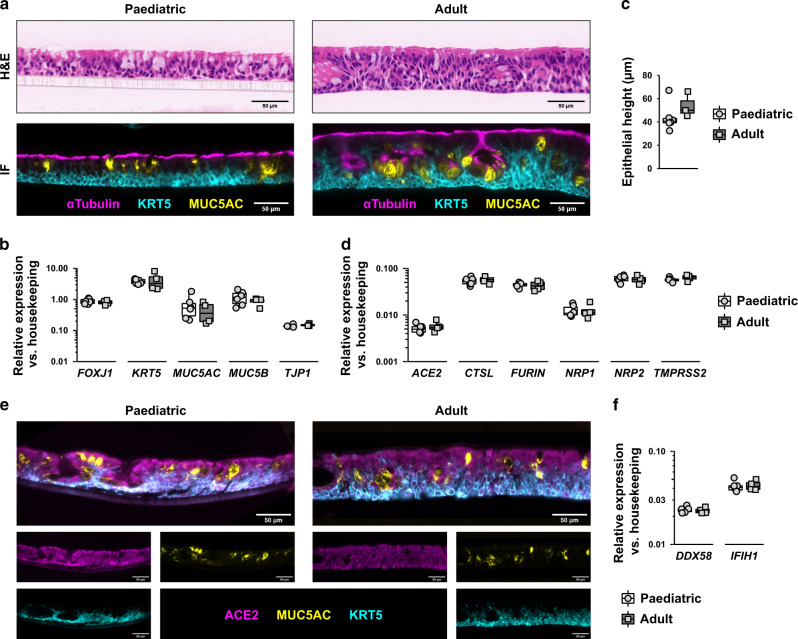
Bronchial epithelial cells from paediatric and adult donors displayed comparable cellular composition and SARS-CoV-2 entry factor expression. **a** Representative images of H&E or immunofluorescence staining of uninfected bronchial epithelial cells from paediatric and adult donors. Immunofluorescence: Ciliated cells (acetylated alpha tubulin; magenta), basal cells (KRT5; cyan) and goblet cells (MUC5AC; yellow). Length of scale bar: 50 µm. **b** Gene expression analysis by qPCR from uninfected bronchial epithelial cells. **c** Epithelial height measured in cross-section of fixed bronchial epithelial cells from paediatric or adult donors. **d** Gene expression analysis by qPCR from uninfected bronchial epithelial cells. **e** Representative images of staining for KRT5 (cyan), ACE2 (magenta) and MUC5AC (yellow) in uninfected bronchial cells from paediatric and adult donors. Size of scale bar: 50 µm. **f** Gene expression analysis by qPCR from uninfected bronchial epithelial cells.

When cultured at air–liquid interface and fully differentiated, epithelial cells from both age groups showed similar morphology, characterised by the presence of basal cells (KRT5^+^), goblet cells (MUC5AC^+^) and ciliated cells (acetylated alpha-tubulin^+^, Fig. 1a), and no differences in the expression of genes related to epithelial cell subsets or tight junction formation (*TJP1*) were observed between the groups (Fig. 1b). Epithelial height varied slightly between donors of both groups (Fig. 1c). We also examined the expression levels of *ACE2* and *TMPRSS2*, two cell surface proteins required for SARS-CoV-2 entry, as well as further SARS-CoV-2 entry-related genes (*CTSL*, *FURIN*, *NRP1* and *NRP2*), and found all of them to be expressed at comparable levels between groups (Fig. 1d), suggesting similar susceptibility to SARS-CoV-2 entry. ACE2 expression was further confirmed at the protein level by immunofluorescence staining, where it was detected across the entire cell layer (Fig. 1e), indicating its expression by ciliated cells, basal cells and at least a proportion of goblet cells (Supplementary Fig. 2). Lastly, we measured expression levels of *DDX58* (encoding RIG-I) and *IFIH1* (encoding MDA5), two PRRs proven (MDA5) or speculated (RIG-I) to be important in the recognition of SARS-CoV-2^20,35,39^. While both genes were detectable in uninfected cells, there were no differences in expression levels between paediatric and adult cells (Fig. 1f). Overall, our analysis did not reveal any intrinsic differences between paediatric and adult cells that would be expected to result in disparate susceptibility to SARS-CoV-2 entry or capacity for viral recognition.

### Age did not distinguish epithelial susceptibility and immune responses to SARS-CoV-2, which were minimal compared to H1N1

**Fig. 2:**
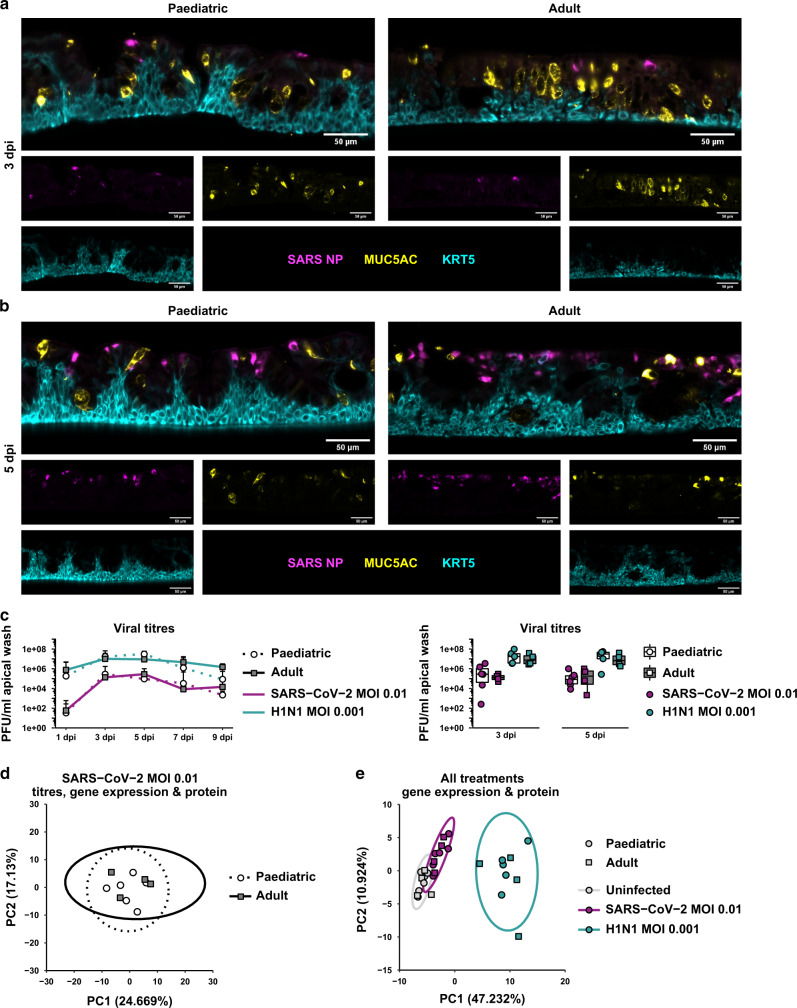
Age did not distinguish epithelial susceptibility and immune responses to SARS-CoV-2, which were minimal compared to H1N1. **a**, **b** Representative images of immunofluorescence staining for SARS-CoV-2 nucleocapsid protein (NP) (magenta), MUC5AC (yellow) and KRT5 (cyan) in SARS-CoV-2-infected cells at 3 (A) or 5 (B) days post-infection (dpi). Size of scale bar: 50 µm. **c** Viral titres in apical wash of epithelial cells infected with SARS-CoV-2 or H1N1 as determined by plaque assays. Line graph in (A) shows median + confidence interval. **d** Principal component analysis of SARS-CoV-2-infected cells based on 121 variables (viral titres, expression of 28 genes and concentration of 2 proteins across up to five time points). Each data point represents one donor. **e** Principal component analysis of epithelial cells based on 98 variables (expression of 28 genes and concentration of 2 proteins across up to five time points). Each data point represents one treatment of one donor.

To compare viral replication between age groups and viruses, as well as to study the resultant cellular immune responses, bronchial epithelial cells were infected with SARS-CoV-2 (SARS-CoV-2/England/IC19/2020; MOI 0.01) or Influenza A H1N1 virus (A/England/195/2009; MOI 0.001) (Supplementary Fig. 1D). To investigate which epithelial subtypes were infected by SARS-CoV-2, we performed immunofluorescence staining for SARS-CoV-2 nucleocapsid protein (NP) in cell layers at 3 or 5 dpi (Fig. 2a, b). The majority of SARS-CoV-2 NP signal did not co-localise with either KRT5 or MUC5AC signal. These data, coupled with the apical location of the SARS-CoV-2 NP staining suggest that ciliated, not basal or goblet cells, were the primary site of SARS-CoV-2 infection. We also noticed a clear increase in the number of infected cells between 3 and 5 dpi (Fig. 2a, b), indicative of continued replication of the virus, which was further confirmed by performing plaque assays on apical washes. Release of infectious SARS-CoV-2 particles from infected cells peaked at around 3–5 dpi and remained detectable throughout the duration of the experiment (9 dpi; Fig. 2c). In line with comparable expression of SARS-CoV-2 entry and pattern recognition receptors, we did not observe any differences between paediatric and adult cells regarding viral replication at any stage during the infection (Fig. 2c). Notably, levels of infectious particles released from H1N1-infected cells were several log-fold higher than from SARS-CoV-2-infected cells, especially at the earliest time point (1 dpi), indicating a better ability of H1N1 to replicate in bronchial epithelial cell cultures compared to SARS-CoV-2, at least for these donors and virus strains.

**Fig. 3:**
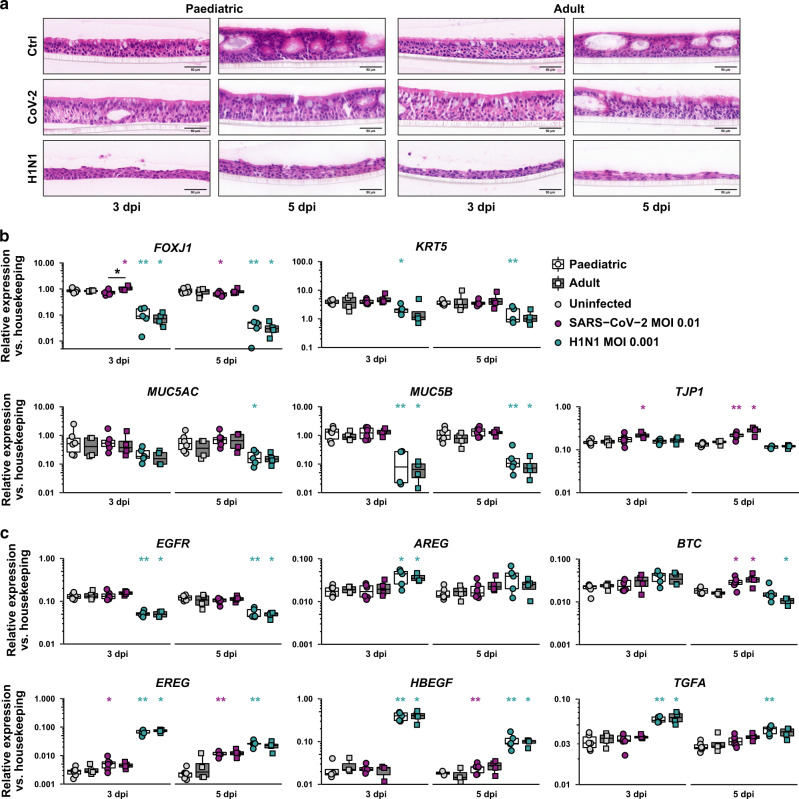
Infection of epithelial cells with SARS-CoV-2 did not alter gross cellular morphology. **a** Representative images of H&E staining of paediatric or adult bronchial epithelial cells infected with SARS-CoV-2 (CoV-2), H1N1 or treated with medium (ctrl) at 3 and 5 days post-infection (dpi). **b**, **c** Gene expression analysis by qPCR of infected bronchial epithelial cells. Coloured asterisks indicate statistically significant changes from uninfected conditions. Black bar and asterisk indicate statistically significant differences between paediatric and adult cells. Statistics: Mann–Whitney test. *p < 0.05; **p < 0.01.

**Fig. 4:**
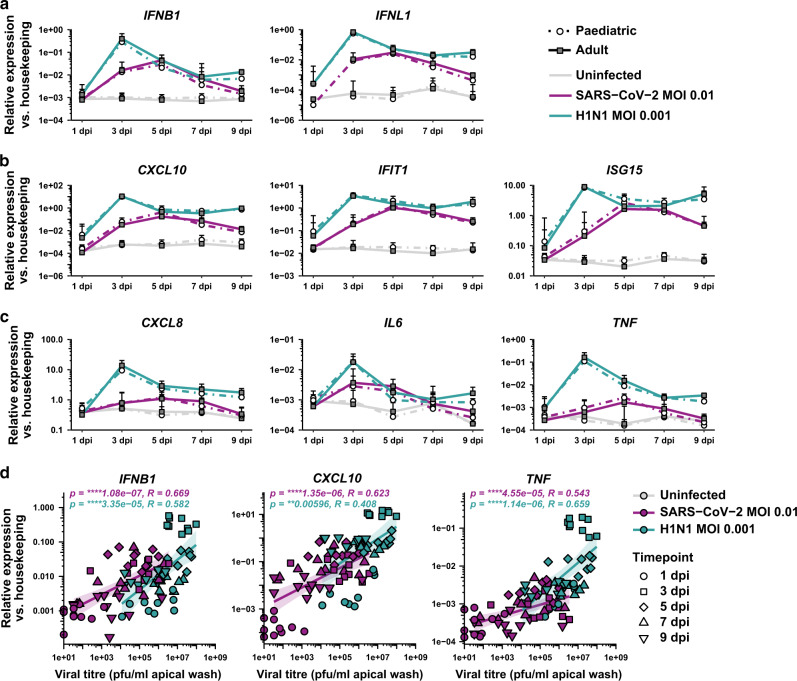
Infection with SARS-CoV-2 elicited antiviral and pro-inflammatory transcriptional programmes, which were significantly weaker than with H1N1 infection. **a**–**c** Gene expression analysis by qPCR of infected bronchial epithelial cells. Data are shown as median + confidence interval. **d** Spearman correlation between viral titres and gene expression of *IFNB1* or *TNF* of paediatric and adult cells infected with SARS-CoV-2 or H1N1. Correlation coefficients and *p* values are shown for each treatment individually (purple, SARS-CoV-2; teal, H1N1).

**Fig. 5:**
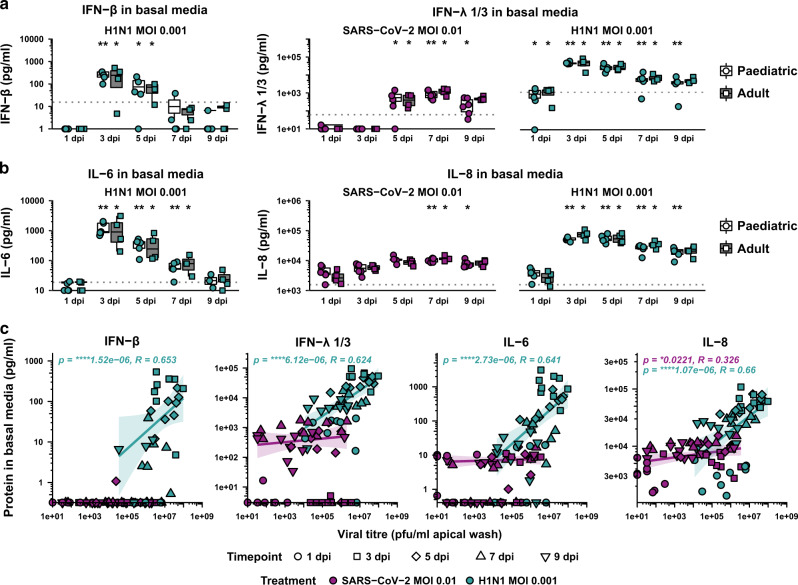
Protein production by epithelial cells in response to SARS-CoV-2 infection was minimal compared to H1N1. **a**, **b** Concentrations of indicated proteins in the basal media from epithelial cells infected with SARS-CoV-2 or H1N1. Dotted lines denote lower limit of detection. Asterisks denote statistically significant change from uninfected controls. Statistics: Mann–Whitney test. *p < 0.05; **p < 0.01. **c** Spearman correlation between viral titres and protein concentrations in basal media of cells paediatric and adult cells infected with SARS-CoV-2 or H1N1. Correlation coefficients and p values are shown for each treatment individually (purple, SARS-CoV-2; teal, H1N1).

In addition to viral titres, we also compared immune responses to SARS-CoV-2 infection between paediatric and adult cells (Figs. 3–5). Principal component analysis (PCA) of SARS-CoV-2-infected cells based on 121 variables measured in this study, comprising viral titres, gene expression levels and concentrations of secreted proteins at five different time points, showed a complete overlap between paediatric and adult donors (Fig. 2d), implying that in our subjects, age is unlikely to be a driver of heterogeneity between donors. Our data may suggest that paediatric and adult airway epithelial cells were indistinguishable in their response to infection with SARS-CoV-2 within our experimental setup. However, the inability to detect differences between age groups may also be explained by the limited sample size. We also employed PCA to compare all experimental conditions across the gene expression and protein data collected in this study (Fig. 2e). This analysis revealed stark differences between SARS-CoV-2-infected cells, which partly overlapped with uninfected cells, and H1N1-infected cells, which displayed a clear separation from the other conditions, suggesting a much greater ability of H1N1 to evoke epithelial cell responses compared to SARS-CoV-2.

### Infection of epithelial cells with SARS-CoV-2 did not alter gross cellular morphology

Given the considerable divergence between H1N1- and SARS-CoV-2-infected cells brought to light by the principal component analysis, we sought to characterise the disparate effects of the two viruses on bronchial epithelial cells in more detail. We used a combination of traditional haematoxylin and eosin staining and gene expression analysis of cell layers to assess the consequences of infection on overall morphology and epithelial integrity. While infection with H1N1 severely affected the structure and height of the cell layer (Fig. 3a) and further led to reduced expression of ciliated, basal and goblet cell genes (Fig. 3b), SARS-CoV-2 did not alter gross cell morphology and even resulted in increased levels of *TJP1*. We also measured the expression of the epidermal growth factor receptor (*EGFR*), a receptor thought to be important for airway epithelial repair^40^, as well as its seven ligands: amphiregulin (*AREG*), betacellulin (*BTC*), epidermal growth factor (*EGF; not shown*), epigen (*EPGN*; not detected), epiregulin (*EREG*), heparin-binding EGF-like (*HBEGF*) and transforming growth factor-alpha (*TGFA*). While infection with both viruses led to significant increases in ligand expression, the effects of H1N1 infection were wider-ranging and of greater magnitude (Fig. 3c). Taken together, these data suggest that, in comparison to infection with H1N1, SARS-CoV-2 only caused minimal damage and subsequent repair of the epithelium.

### Infection with SARS-CoV-2 elicited antiviral and pro-inflammatory transcriptional programmes, which were significantly weaker than with H1N1 infection

We next considered the epithelial immune response. Infection with SARS-CoV-2 strongly induced the expression of type I (*IFNB1*) and type III (*IFNL1*) IFNs as well as several ISGs, although peak expression levels resulting from SARS-CoV-2 infection were lower than after H1N1 infection (Fig. 4a, b, Fig S3A). Infection with either virus also resulted in an induction of the pro-inflammatory genes *CXCL8* (encoding IL-8), *IL6* and *TNF*, albeit to a lesser extent in SARS-CoV-2-infected cells (Fig. 4c). Importantly, however, expression levels of interferon signalling genes as well as pro-inflammatory genes strongly correlated with infectious titres of both viruses in apical washes (Fig. 4d, Supplementary Fig. 3A, B), suggesting that the milder inflammatory response observed in SARS-CoV-2-infected cells may result from lower viral replication in these cells. Apart from slightly elevated expression of *IFNB1* and *TNF* in H1N1-infected adult compared to paediatric cells at 9 dpi (*p* = 0.016), there were no differences between the age groups.

### Protein production by epithelial cells in response to SARS-CoV-2 infection was minimal compared to H1N1

In addition to the transcriptional response, we also measured proteins secreted by epithelial cells infected with either SARS-CoV-2 or H1N1. As expected, H1N1 infection resulted in release of IFN-β, IFN-λ, IL-6 and IL-8 into the basal media (Fig. 5a, b), of which IL-8 alone was also present in media from uninfected cells (Supplementary Fig. 4A). In contrast, only IFN-λ and IL-8 were detected in media from SARS-CoV-2-infected cells, at levels approximately 5- to 40-fold lower than after H1N1 infection (Fig. 5a, b). To address whether elevated interferon levels in the basal media of H1N1-infected cells might be secondary to increased leakage of apically released protein as a result of damage to the epithelial layer, we also measured levels of IFN-β and IFN-λ in apical washes of infected cells (Supplementary Fig. 4B). Apical release of both interferons in response to SARS-CoV-2, while detectable, fell short of levels measured after H1N1, indicating an overall diminished ability of SARS-CoV-2 to elicit antiviral or pro-inflammatory protein production in bronchial epithelial cells from healthy individuals. In line with this, basolateral protein release after H1N1 infection strongly correlated with viral titres, whereas we found no or only weak correlation between titres and measured protein levels from SARS-CoV-2-infected cells (Fig. 5c)This finding was further supported when levels of 105 cytokines and chemokines were measured in pooled basal media from infected paediatric (*n* = 4) or adult (*n* = 4) epithelial cells using a membrane-based protein array, where infection with SARS-CoV-2 had little effect on the proteins detected, whereas H1N1 induced a wide range of analytes (Supplementary Fig. 4B). Overall, these data confirm that, when compared to H1N1, SARS-CoV-2 had a diminished potential to affect epithelial morphology or induce interferon and pro-inflammatory responses at both mRNA and protein level, which may at least in part result from its relatively reduced replication in healthy bronchial epithelial cells.

### Infection of bronchial epithelial cells with H1N1 resulted in prolonged upregulation of SARS-CoV-2 entry-related genes independent of viral titres

**Fig. 6:**
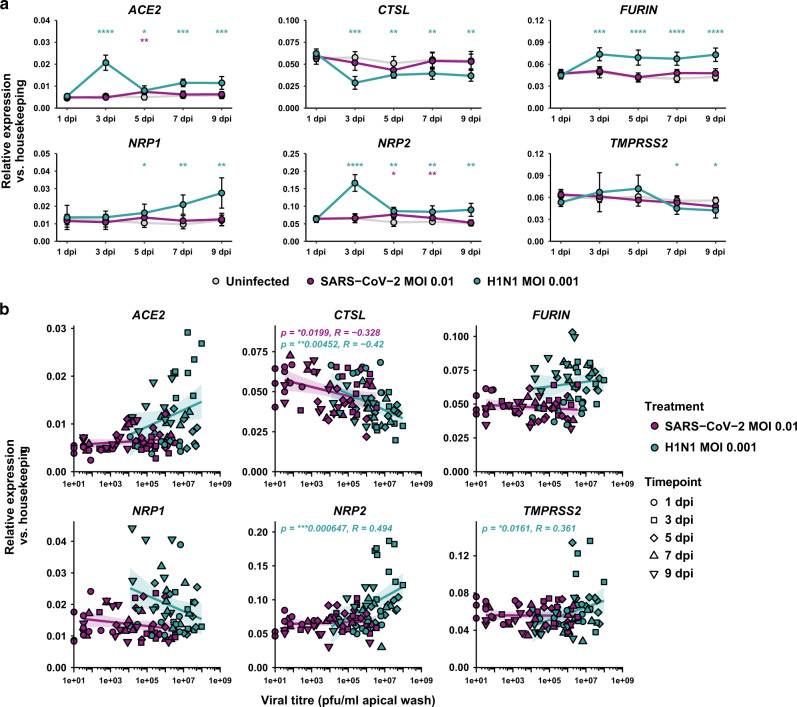
Infection of bronchial epithelial cells with H1N1 resulted in prolonged upregulation of SARS-CoV-2 entry-related genes independent of viral titres. **a** Gene expression analysis by qPCR of infected bronchial epithelial cells. Data shown as median ± confidence interval of paediatric (*n* = 5–6) and adult (*n* = 4) donors pooled together. Asterisks denote statistically significant change from uninfected control. Statistics: Mann–Whitney test. *p < 0.05; **p < 0.01; ***p < 0.001; ****p < 0.0001. **b** Spearman correlation between viral titres and gene expression of indicated genes in cells infected with SARS-CoV-2 or H1N1. Correlation coefficients and *p* values are shown for each treatment individually (purple, SARS-CoV-2; teal, H1N1).

Finally, we aimed to investigate whether viral infection can alter the expression of genes involved in SARS-CoV-2 cell entry and could thus potentially affect susceptibility to subsequent infections. As we did not observe any differences between paediatric and adult cells, both groups were pooled for this analysis. While the expression of entry-related genes was largely unaffected by SARS-CoV-2 infection, cells infected with H1N1 showed significantly altered expression of all six genes, with *ACE2*, *FURIN*, *NRP1* and *NRP2* being significantly upregulated compared to uninfected controls at 9 dpi (Fig. 6a). We found *FURIN* to be of special interest, as its expression was induced early following H1N1 infection and remained consistently elevated through the experiment. *FURIN* levels did not correlate with viral titres (Fig. 6b), suggesting that expression of this gene is likely controlled by mechanisms distinct from the antiviral and pro-inflammatory response. Similarly, *NRP1* expression levels were unchanged at 3 dpi but increased significantly and continuously from 5 dpi. Together, these data indicate that H1N1 infection resulted in sustained upregulation of SARS-CoV-2 entry-related genes in bronchial epithelial cells even after viral titres decreased. If similar events took place in vivo, they may result in an increased risk of SARS-CoV-2 infection in patients recently recovering from influenza.

## Discussion

There are growing efforts to understand why children typically develop less severe COVID-19 disease than adults, which is in contrast to findings from other respiratory viruses that have resulted in pandemics, such as H1N1. The mechanisms underlying these discrepancies in COVID-19 severity remain poorly understood. Here, we hypothesised that intrinsic differences in the responses of healthy paediatric and adult bronchial epithelial cells to infection with SARS-CoV-2 could explain the differences observed according to age. In order to understand whether susceptibility to infection and immune responses were specific for SARS-CoV-2, we compared it to H1N1. Our characterisation of viral replication and epithelial immune responses following SARS-CoV-2 infection of differentiated bronchial epithelial cells from healthy children and adults showed indistinguishable infection susceptibility and immune responses between paediatric and adult cells. However, we acknowledge that a larger sample size may have revealed age-related differences. SARS-CoV-2 infection induced a robust transcriptional interferon response, which was delayed compared to H1N1 infection, in line with slower viral replication of SARS-CoV-2. Protein production and repair responses were also much lower in SARS-CoV-2- compared to H1N1-infected cells, and only H1N1 infection resulted in changes to the morphology and composition of the epithelial layer.

Mounting evidence cements the central role of type I IFNs in the generation of effective responses to SARS-CoV-2 infection, including observations of higher prevalence of loss-of-function mutations or auto-antibodies interfering with type I IFN signalling in patients with life-threatening COVID-19^24,25^. The benefit of interferon signalling has also been demonstrated in primary airway epithelial cells in vitro, where the application of either type I and III IFNs before or early after infection with SARS-CoV-2 was shown to result in reduced viral burden in both nasal and bronchial epithelial cells^35,37,38^. A recent study comparing the upper airway transcriptome of healthy uninfected and SARS-CoV-2-infected children and adults using single-cell RNA sequencing (scRNA-seq) of nasal swabs reported higher expression of *IFIH1* and *DDX58* in epithelial cells from children, both at baseline and during the early phase of SARS-CoV-2 infection^20^. *IFIH1* and *DDX58* encode MDA5 and RIG-I, respectively, two PRRs important in the induction of interferon signalling in response to infection with viruses, of which MDA5 was specifically shown to contribute to sensing of SARS-CoV-2^35,39^. As such, a higher expression of the relevant PRRs needed for the induction of IFN signalling might potentially result in faster and stronger antiviral signalling upon infection. In line with this, a study comparing nasopharyngeal swabs from 12 children and 27 adults admitted to the emergency department with SARS-CoV-2 infection found increased levels of interferons, ISGs and pro-inflammatory cytokines in nasal fluids from children compared to adults. This heightened immune response signature aligned with better clinical outcomes^21^, suggesting children may indeed be more poised to mount an inflammatory response to SARS-CoV-2 than adults.

When we compared the expression of *IFIH1* and *DDX58* between bronchial epithelial cell cultures collected from healthy children and adults and cultured at air–liquid interface, we did not observe any differences in PRR expression or the ability of the cells to mount an antiviral interferon response to infection with SARS-CoV-2. We cannot exclude that a comparison of nasal rather than bronchial epithelial cells or an increase in sample size would have revealed differences between groups, and potentially even in the ability to control SARS-CoV-2 replication. However, it is more likely that the increased PRR expression levels in children are not an intrinsic feature of paediatric airway epithelia, but rather a result of environmental influences. An increased antiviral interferon signature, including increased expression of PRRs, could be the result of recent respiratory virus infections, which are more common in children compared to adults^41^. The effects of infection history are not limited to the airway epithelium; a study has reported the existence of SARS-CoV-2 spike glycoprotein (S)-binding antibodies in the serum of individuals not previously exposed to the virus or the vaccine^42^. These pre-existing antibodies were most commonly found in children and adolescents and are thought to be cross-reactive immunological memory originally mounted in response to other seasonally circulating human coronaviruses, which are more prevalent in children compared to adults^43,44^.

Allowing for a relatively modest sample size, our preliminary findings suggest that disparate disease severity of COVID-19 between children and adults may not be the result of cell-intrinsic differences between paediatric and adult airway epithelia. This interpretation warrants further investigation in future studies that should aim to increase the sample size and include epithelial cells from a wider age range of subjects. Even so, it is unlikely that infection history alone is the deciding factor for disease severity. It is thus important to also consider the contribution of other anatomical sites and cell types. While our model is ideally suited for the detailed characterisation of the normal airway epithelial response to SARS-CoV-2 infection, it is unable to reflect the progression of SARS-CoV-2 to the alveoli, a process associated with the development of severe disease^27^. Another limitation of the present study is the inability to demonstrate how airway epithelial cells interact with other structural and immune cells and how this may shape the response to infection, including viral clearance. The use of scRNA-seq in samples from individuals infected with SARS-CoV-2 has provided some insights into these interactions in the context of diverging COVID-19 susceptibility and severity between children and adults^20,45^. In addition to differences in epithelial expression of pattern recognition receptors, as discussed above, these studies demonstrated augmented frequencies of diverse lymphocyte populations, such as innate lymphoid cells and various T cell subsets, in children. Ultimately, it is likely that many factors, coupled with their interaction with each other, influence the disparate disease severity in individuals infected with SARS-CoV-2.

A stark finding of our study was the pronounced difference between infection with SARS-CoV-2 versus H1N1. SARS-CoV-2 showed much lower release of infectious particles compared to H1N1, suggesting a diminished ability to replicate in airway epithelia. This may explain the longer incubation period of around 6 days for SARS-CoV-2^46^ compared to the much shorter incubation period of 1–4 days for H1N1^47^ in infected individuals. Interestingly, we found no evidence of damage or overt pro-inflammatory responses after SARS-CoV-2 infection, both of which were evident in H1N1-infected cells. This is surprising considering both viruses have similar spectra of disease severity and case fatality rates are estimated to be even higher for COVID-19 compared to influenza^48^. This suggests that, in contrast to H1N1, the epithelial response to SARS-CoV-2 may be less consequential to disease severity.

Early studies of SARS-CoV-2 infection in ALI cultures of bronchial epithelial cells reported an inability of the cells to mount an interferon response to the virus^36,37^. In contrast, various other reports have more recently demonstrated induction of interferon signalling upon SARS-CoV-2 infection in primary nasal, tracheal and bronchial epithelial cells^34,35,38,39,49,50^. Of note, interferon and ISG induction was primarily reported during later stages of the infection (around 2–4 dpi) and was delayed and of lower magnitude when compared to infection with Influenza A H1N1^38^ or H3N2^34^, a finding which is replicated in our data. This delay in the onset of the interferon response was hypothesised to result from the ability of SARS-CoV-2 to subvert early interferon induction. Several viral evasion strategies of SARS-CoV-2 have been brought to light. These include the use of double-membrane vesicles for viral RNA synthesis^51^ and the ability of non-structural proteins and other viral proteins to inhibit interferon induction and signalling^52,53^ as well as interference with protein translation^54–57^. However, our data indicate a strong correlation between levels of infectious viral particles released into the apical wash and gene expression of *IFNB1*, *IFNL1* and several ISGs following infection with either SARS-CoV-2 or H1N1, suggesting that reduced ability of SARS-CoV-2 to replicate in bronchial epithelial cells, rather than its evasion strategies, is the major driver for the delayed interferon response at transcriptional level. Nevertheless, the minimal protein release from SARS-CoV-2-infected cells, both apically and basolaterally, did not match the robust interferon induction elicited at transcriptional level, suggesting that viral interference with protein translation may have occurred.

Lastly, we observed that infection with H1N1 resulted in prolonged upregulation of the SARS-CoV-2 entry-related genes *FURIN* and *NRP1* that was not linked to viral titres. Both furin and neuropilin 1 have been shown to potentiate SARS-CoV-2 infectivity^11,13,14^. It may be that an increased expression of these proteases renders individuals recovering from flu more susceptible to SARS-CoV-2 infection. Co-infection experiments in primary airway epithelial cells, while beyond the scope of the present study, will be instrumental in supporting this hypothesis. In their absence, however, others have shown influenza A pre-infection to increase the infectivity of SARS-CoV-2 in vitro^58^ and mice infected with influenza and subsequently SARS-CoV-2 displayed enhanced disease susceptibility and pathology compared to those infected with SARS-CoV-2 alone^58,59^. Importantly, a study evaluating 19,256 individuals tested for both influenza and SARS-CoV-2 in England identified a greater risk of death in patients with co-infection compared to those infected with either virus alone^60^. These findings underscore the absolute need for public policy to urge immunisation against influenza as well as COVID-19.

Overall, we demonstrated that intrinsic differences in bronchial epithelial cells from children and adults did not explain the discrepancies in COVID-19 disease susceptibility and severity seen between those groups. However, when comparing two viruses that have both resulted in pandemics, there was a stark difference in epithelial damage and immune responses. In addition to a previously described delay in induction of interferon signalling, which we were able to link to delayed replication, SARS-CoV-2 caused no damage to the epithelial layer and displayed an overall diminished capacity to evoke epithelial immune responses at both mRNA and protein level. In contrast, H1N1 infection resulted in rapid viral replication, robust epithelial damage, altered morphology of the cell layer and markedly greater immune responses.

## Methods

### Patient recruitment and sampling

Ethical approval for sample collection was granted by the Research Ethics Committee (17/LO/0013 and 15/SC/0101) and all patients or parents provided informed written consent.

Children aged 2–5 with no history of wheezing or other respiratory pathology (*n* = 6) (Table 1) were recruited as part of the Breathing Together study as previously described^61^. Bronchial epithelial cells were collected by blind endobronchial brushing from children undergoing anaesthesia for an elective surgical procedure requiring clinically indicated intubation, and nasal epithelial cells were collected from the same donors as previously described^61^. Healthy adults (*n* = 4) (Table 1) were recruited for bronchoscopies, during which bronchial brushes were taken from the right mainstem bronchus. Brushes were collected in DMEM media containing penicillin (100 U/ml) and streptomycin (100 µg/ml; all Gibco). Cells were detached by agitation and then expanded for two passages in Airway Epithelial Cell Growth Medium (Promocell) containing 100 µg/ml Primocin (InvivoGen) in flasks coated with 30 µg/ml (bronchial) or 100 µg/ml (nasal epithelial cells) bovine Type I Collagen Solution (BioMatrix) before cryopreservation.

### Air–liquid interface culture of epithelial cells

Cryopreserved cells were expanded in Airway Epithelial Cell Growth Medium containing penicillin and streptomycin before seeding onto collagen-coated (30 µg/ml type I collagen) 24-well Transwell inserts (polyethylene terephthalate membrane with 0.4 µm pores; Corning) at a density of 3 × 10^4^ per insert. A 1:1 mix of Airway Epithelial Growth Medium (without the Triiodo-L-thyronine supplement) containing penicillin and streptomycin and DMEM (high glucose, GlutaMAX™, pyruvate; Gibco) containing penicillin and streptomycin, 25 mM HEPES (Gibco) and 1.5 µg/ml BSA (Sigma) was used for maintenance of the cells during seeding and submerged phase of the culture, with 200 µl and 500 µl of medium in the apical and basal compartment, respectively. Medium was changed every 2–4 days.

Upon confluency, apical medium was removed, and the basal medium was replaced with PneumaCult™-ALI Medium containing 0.0004% Heparin, 0.48 µg/ml hydrocortisone (all Stemcell) and penicillin and streptomycin. Basal medium was refreshed every 2–4 days until cells were fully differentiated (4–9 weeks) and where necessary, mucus was removed by addition of 100 µl PBS to the apical compartment for 5 min before removal.

At least 48 h before infection, hydrocortisone was withdrawn from the media and cells were cultured without hydrocortisone for the entirety of the experiment. Apical washes and basal media were collected 24 hours after infection and then every 48 h. For apical washes, 200 µl of PBS were added to the apical compartment and collected after 10 min of incubation at room temperature.

### Viral stocks, infection, and determination of viral titres

All work involving the use of live SARS-CoV-2 or H1N1 was carried out in the Containment Level 3 (CL3) or Containment Level 2 (CL2) laboratories (St. Mary’s, Imperial College London), respectively. Stocks of SARS-CoV-2 (SARS-CoV-2/England/IC19/2020) and Influenza H1N1 (A/England/195/2009) were propagated in Vero-E6 or MDCK cells, respectively.

Prior to infection, the apical compartment was washed once with PBS and the basal media was replaced. A cell density of 2.5 × 10^5^ cells per insert was assumed. 2.5 × 10^3^ PFU SARS-CoV-2 (MOI 0.01), 2.5 × 10^2^ PFU H1N1 (MOI 0.001) or serum-free DMEM were added to the apical compartment in a total volume of 100 µl and cells were incubated for 1 hr at 37 °C/5% CO_2_ before apical liquid was removed and cells were returned to the incubator.

Viral titres of SARS-CoV-2 and H1N1 in apical washes were determined using plaque assays in Vero-E6 or MDCK cells, respectively.

### Protein mediator analysis

All samples were irradiated prior to analysis to allow work to be conducted outside of CL2 or CL3 facilities. Levels of IFN-β, IFN-λ 1/3, IL-6 and IL-8 were measured in basal media or apical washes from at least three inserts per condition using DuoSet ELISA kits (R&D Systems) according to manufacturer’s recommendation. Levels of 105 cytokines were measured in pooled basal media from four paediatric or adult donors, respectively, using the Proteome Profiler Human XL Cytokine Array Kit (R&D Systems) according to manufacturer’s recommendation.

### Gene expression analysis

Total RNA was extracted from two Transwell inserts per condition using the Maxwell® RSC simplyRNA Blood kit on the Maxwell® RSC instrument (both Promega) and transcribed into cDNA using the High-Capacity cDNA Reverse Transcription kit (Applied Biosystems). Expression of genes of interest relative to the expression of two housekeeping genes (*ATP5B, YWHAZ*) was determined using TaqMan™ Fast Advanced reagents on a ViiA 7 Real-Time PCR system (both Applied Biosystems). TaqMan™ assay IDs are listed in Table 2.

**Table 2:** TaqMan™ assays used for gene expression analysis.

Gene	Assay ID	Gene (cont.)	Assay ID (cont.)
*ACE2* (full-length isoform)	Hs00222343_m1	*IFIT1*	Hs00356631_g1
*AREG*	Hs00950669_m1	*IFNB1*	Hs01077958_s1
*ATP5B* (housekeeping)	Hs00969569_m1	*IFNL1*	Hs00601677_g1
*BTC*	Hs01101204_m1	*IL6*	Hs00174131_m1
*CTSL*	Hs00964650_m1	*ISG15*	Hs01921425_s1
*CXCL10*	Hs01124252_g1	*KRT5*	Hs00361185_m1
*CXCL8*	Hs00174103_m1	*MUC5AC*	Hs01365616_m1
*DDX58*	Hs01061436_m1	*MUC5B*	Hs00861595_m1
*EGF*	Hs01099990_m1	*NRP1*	Hs00826128_m1
*EGFR*	Hs01076090_m1	*NRP2*	Hs00187290_m1
*EPGN*	Hs02385424_m1	*TGFA*	Hs00608187_m1
*EREG*	Hs00914313_m1	*TJP1*	Hs01551861_m1
*FOXJ1*	Hs00230964_m1	*TMPRSS2*	Hs01122322_m1
*FURIN*	Hs00965485_g1	*TNF*	Hs00174128_m1
*HBEGF*	Hs00181813_m1	*YWHAZ* (housekeeping)	Hs01122445_g1
*IFIH1*	Hs00223420_m1		

### Histology and immunofluorescence staining

Whole inserts were fixed in 4% (w/v) paraformaldehyde in PBS for 20 min at room temperature. Membranes were excised from inserts and embedded in 4% low melting point agarose (Thermo Scientific) prior to paraffin embedding and sectioning at 4 µm thickness. Paraffin embedding, sectioning and haematoxylin and eosin (H&E) staining were performed by Lorraine Lawrence of the Histology facility at Imperial College London. For immunofluorescence staining, slides were subjected to heat-induced antigen retrieval in 1 mM EDTA, pH 8.0 prior to blocking in PBS containing 10% donkey serum and 0.1% Tween 20 and incubation with antibodies in 1% BSA in PBS at the concentrations indicated in Table 3. In some instances, DAPI (Sigma-Aldrich) was included as a counterstain for nuclei. Images were acquired using the Aperio VERSA slide scanner (Leica) at a maximum magnification of x40. Epithelial height was measured in H&E-stained sections using ImageScope Software (Leica). Fluorescence images were adjusted for contrast and gamma in ImageScope software before export. Fiji software^62^ was used to rotate and crop images, apply pseudocolours and add scale bars. For visualisation of overlap between stains, segmentation was applied on inverted single-channel images using the PHANTAST^63^ plug-in for Fiji and overlapping areas between different colours were identified using the Image Calculator function.

**Table 3:** Antibodies used for immunofluorescence staining.

*Primary antibodies*			
Antibody (clone)	Conjugate	Supplier (catalogue #)	Concentration
ACE-2	–	R&D Systems (AF933)	4.0 µg/ml
Mucin 5AC (EPR16904)	AF555	abcam (ab218714)	5.0 µg/ml
Cytokeratin 5 (EP1601Y)	AF647	abcam (ab193895)	2.5 µg/ml
Goat IgG, polyclonal (isotype)	–	abcam (ab37373)	4.0 µg/ml
SARS Nucleocapsid protein	–	Novus Bio (NB100-56576)	10.0 µg/ml
Alpha Tubulin (6-11B-1)	–	abcam (ab24610)	10.0 µg/ml
Rabbit IgG, Control Antibody	–	Vector Labs (I-1000-5)	10.0 µg/ml

*Secondary antibodies*			
Antibody	Conjugate	Supplier (catalogue #)	Concentration
Donkey anti-mouse IgG	AF488	Invitrogen (A-21202)	10.0 µg/ml
Donkey anti-rabbit IgG	AF594	Invitrogen (A-21207)	10.0 µg/ml
Donkey anti-goat IgG	AF488	Invitrogen (A-11055)	10.0 µg/ml

### Data analysis and statistics

All data processing, analysis and plotting was conducted in R Studio. For statistical analyses, the R stats package was used. When comparing between two groups, exact *p* values were computed using a two-sided Mann–Whitney test and *p* values < 0.05 were considered statistically significant. For correlations, exact *p* values and rank correlation coefficients (rho) were calculated using the Spearman method. Principal component analysis was conducted on centred and scaled data and the first two principal components were plotted. Data was visualised using the ggplot2 package.

## Supplementary information

Supplementary figures
