# Supplementary material for: Distinct airway epithelial immune responses after infection with SARS-CoV-2 compared to H1N1
Source: Mucosal Immunol. 2022 Jul 15;15(5):952–63. doi: 10.1038/s41385-022-00545-4 (PMC9284972; doi:10.1038/s41385-022-00545-4)
Supplement: Supplementary file 1 — Supplementary figures [file 41385_2022_545_MOESM1_ESM.pdf]

Suppl Fig 1

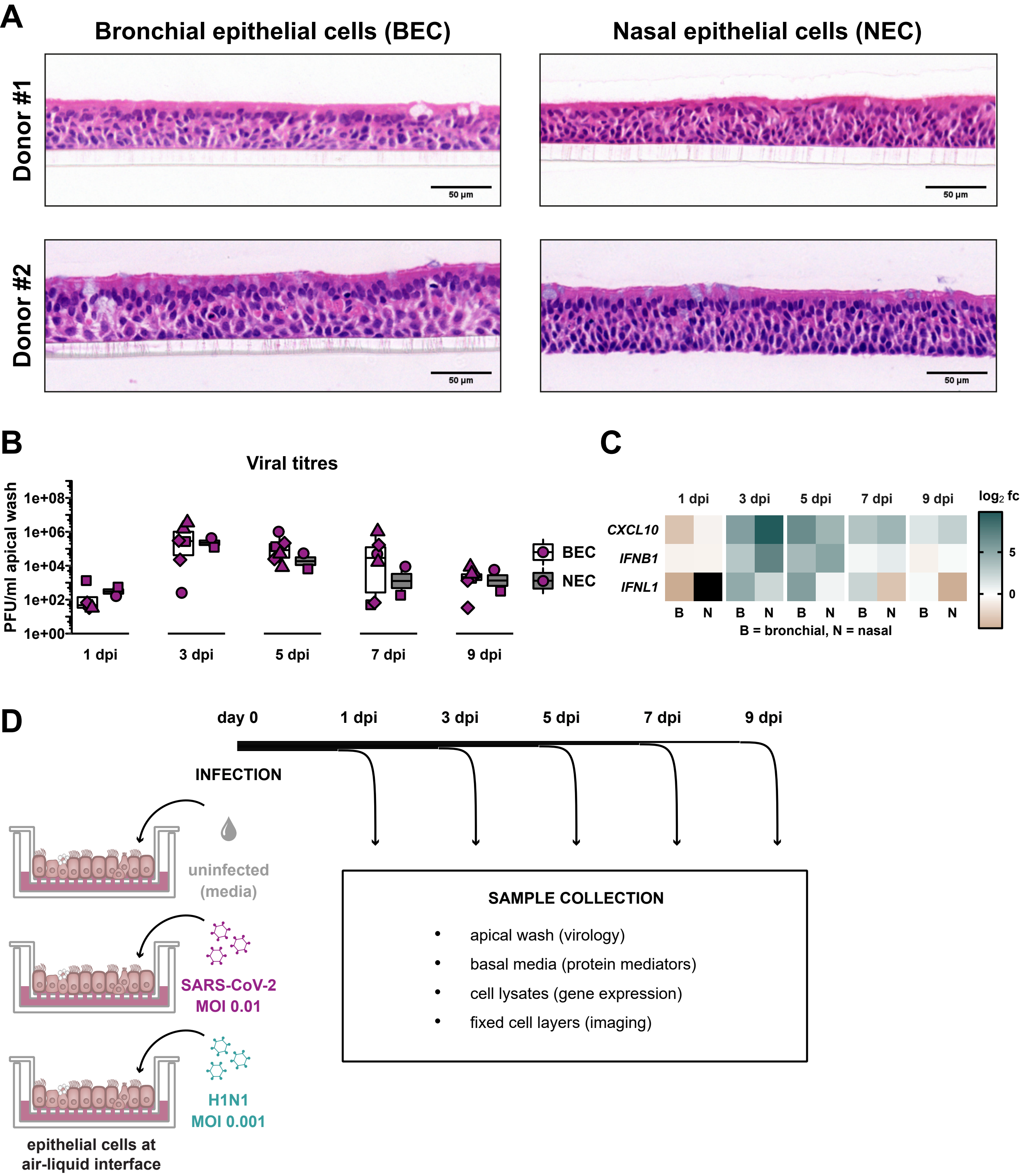

Supplemental Figure 1: Relates to figure 1.

A Representative images of H&E staining of matched uninfected bronchial and nasal epithelial cells from paediatric donors. Length of scale bar: 50 µm.

B Viral titres in apical washes of paediatric epithelial cells infected with SARS-CoV-2 MOI 0.01 as determined by plaque assays. Matched donors are indicated by symbol shape.

C Gene expression analysis by qPCR of paediatric epithelial cells infected with SARS-CoV-2 MOI 0.01. Colour indicates median fold change from uninfected control. Black squares: non-detected.

D Schematic representation of experimental approach. dpi, days post-infection. MOI, multiplicity of infection.

Suppl Fig 2

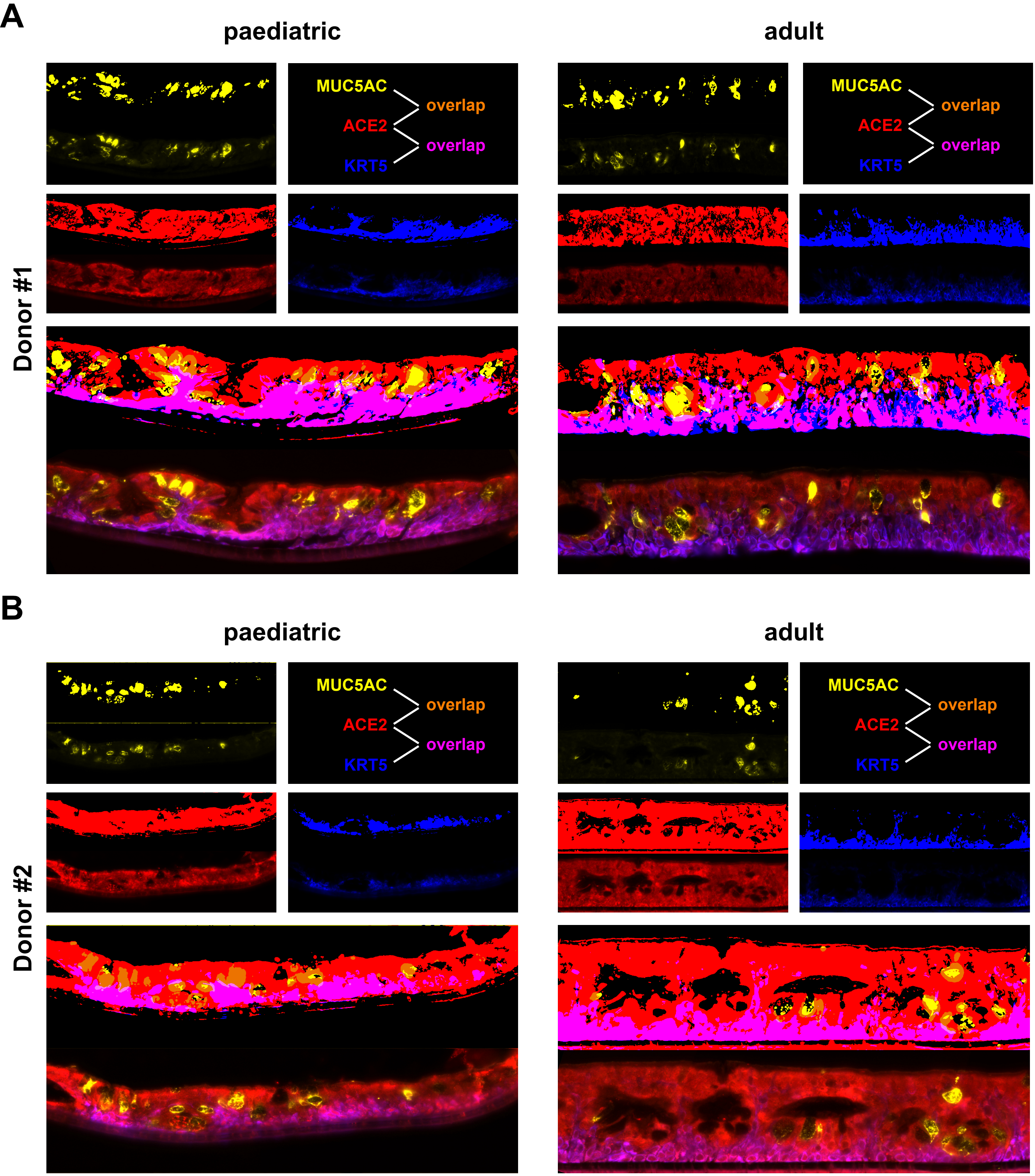

Supplemental Figure 2: Relates to figure 1.

A, B Segmentation of immunofluorescence staining for MUC5AC (yellow), ACE2 (red) and KRT5 (blue) in uninfected epithelial cells from two paediatric and two adult donors, as shown in Fig 1E. Areas where ACE2 staining overlapped with MUC5AC or KRT5 staining are shown in orange and magenta, respectively.

Suppl Fig 3

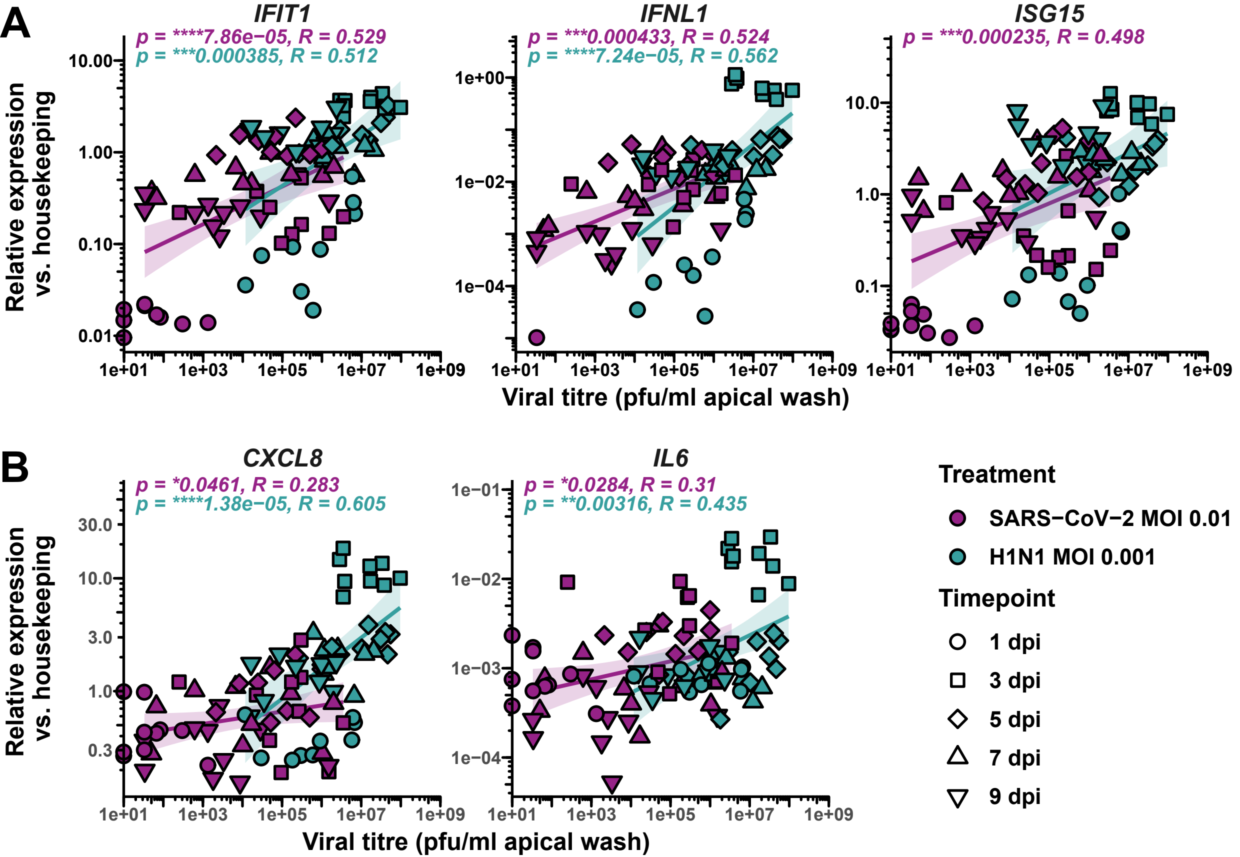

Supplemental Figure 3: Relates to figure 4.

A, B Spearman correlation between viral titres and expression of indicated genes in paediatric and adult cells infected with SARS-CoV-2 or H1N1. Correlation coefficients (R) and p values are shown for each treatment individually (purple, SARS-CoV-2; teal, H1N1).

Suppl Fig 4

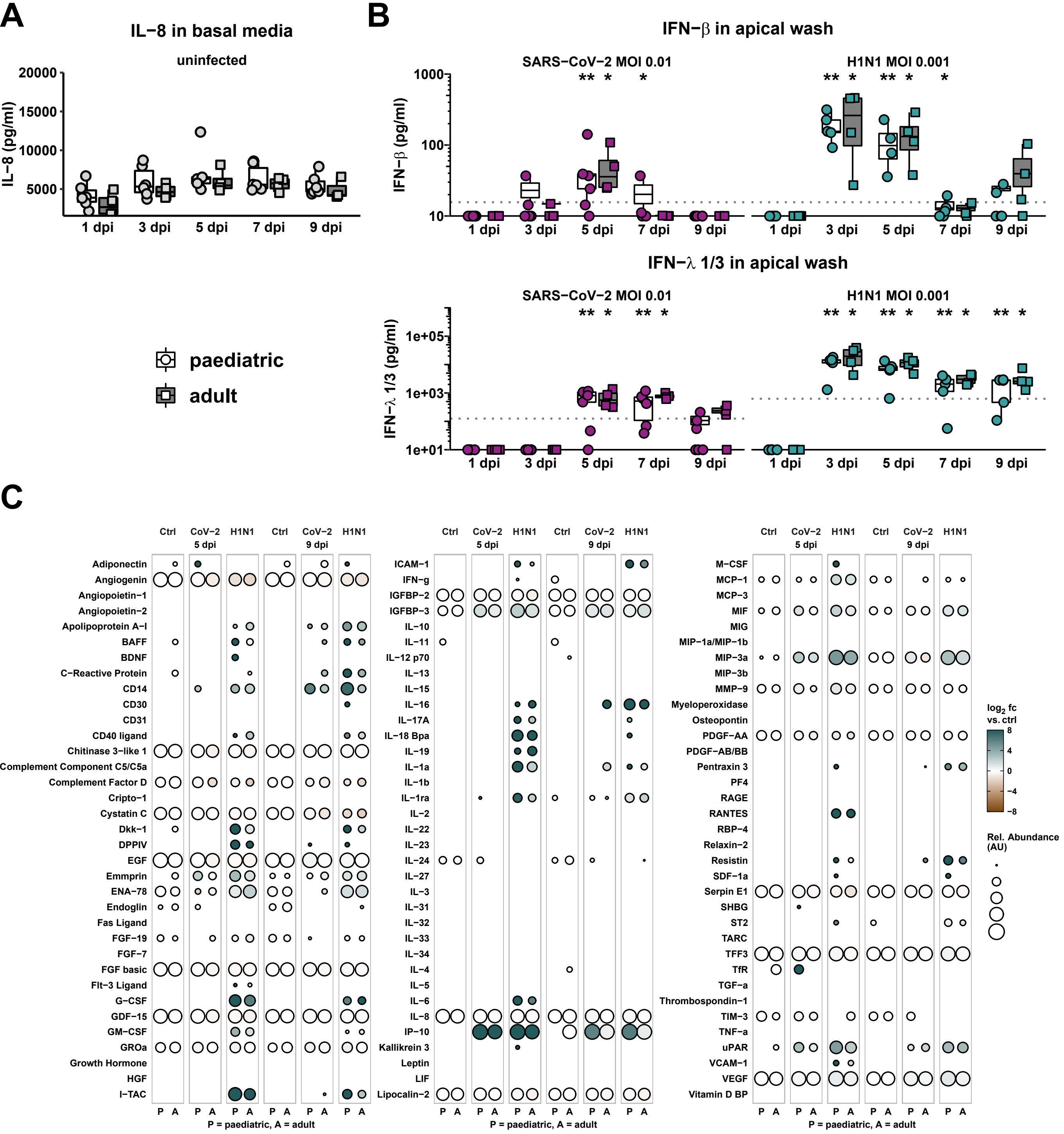

Supplemental Figure 4: Relates to figure 5.

A Concentrations of IL-8 in the basal medium of uninfected cells.

B Concentrations of IFN-β or IFN-λ 1/3 in the apical wash of cells infected with SARS-CoV-2 or H1N1. Asterisks denote statistically significant change from uninfected controls. Statistics: Mann-Whitney test. *, $p < 0.05$; **, $p < 0.01$.

C Relative abundance of 105 proteins in the basal medium of epithelial cells infected with SARS-CoV-2 or H1N1 as measured using a proteome profiler assay. Basal media from four donors per group were pooled and analysed. Size of circles indicates relative abundance and colour indicates fold change from uninfected control. Where mediator was absent in uninfected controls colour of circles was set to dark green.
